# Monkeys Share the Human Ability to Internally Maintain a Temporal Rhythm

**DOI:** 10.3389/fpsyg.2016.01971

**Published:** 2016-12-23

**Authors:** Otto García-Garibay, Jaime Cadena-Valencia, Hugo Merchant, Victor de Lafuente

**Affiliations:** Instituto de Neurobiología, Universidad Nacional Autónoma de MéxicoQuerétaro, Mexico

**Keywords:** rhythm, timing, rhesus, Weber fraction, model of time perception

## Abstract

Timing is a fundamental variable for behavior. However, the mechanisms allowing human and non-human primates to synchronize their actions with periodic events are not yet completely understood. Here we characterize the ability of rhesus monkeys and humans to perceive and maintain rhythms of different paces in the absence of sensory cues or motor actions. In our rhythm task subjects had to observe and then internally follow a visual stimulus that periodically changed its location along a circular perimeter. Crucially, they had to maintain this visuospatial tempo in the absence of movements. Our results show that the probability of remaining in synchrony with the rhythm decreased, and the variability in the timing estimates increased, as a function of elapsed time, and these trends were well described by the generalized law of Weber. Additionally, the pattern of errors shows that human subjects tended to lag behind fast rhythms and to get ahead of slow ones, suggesting that a mean tempo might be incorporated as prior information. Overall, our results demonstrate that rhythm perception and maintenance are cognitive abilities that we share with rhesus monkeys, and these abilities do not depend on overt motor commands.

## Introduction

The ability to estimate time intervals is fundamental to behavior. Motor actions performed outside their intended temporal window often have reduced effectiveness or a complete loss of purpose. However, the mechanisms allowing the brain to time future sensory and motor events are not yet completely understood (Merchant and de Lafuente, 2014). Human, and to a certain extent, monkey subjects can repeatedly tap in synchrony with sensory stimuli (*synchronization*), and they can continue tapping in the absence of external stimuli (*continuation*) (Wing and Kristofferson, 1973; Ivry and Hazeltine, 1995; Zarco et al., 2009; Repp and Su, 2013). The increase in variability of the tapping responses that define time intervals is well described by the generalized Weber's law:

(1) $$\begin{array}{r} \sigma^{2}=k\cdot T^{2}+\sigma_{indep}^{2} \end{array}$$

in which *T* is elapsed time, *k* approaches the square root of the Weber fraction at long elapsed times, and the term $\sigma_{indep}^{2}$ represents a basal variance that does not increase with time (Getty, 1975; Killeen and Weiss, 1987; Gibbon et al., 1997; Bizo et al., 2006; Merchant et al., 2008; Zarco et al., 2009; Laje et al., 2011).

However, the capacity of human and non-human primates to maintain a rhythm in the absence of sensory cues, or a motor action such as tapping, has been less studied (Grahn, 2009; Patel et al., 2009; Fitch, 2013; Repp and Su, 2013). A particularly important question that remains unanswered is whether monkeys are able to perceive and maintain a rhythm in the absence of overt motor actions (Bispham, 2006; Merchant and Honing, 2014). Here we characterize the behavior of human and rhesus subjects in a task in which they have to estimate the tempo of a periodic sensory event and then maintain that rhythm in the absence of movements. We hypothesize that human and monkey subjects share the ability to maintain a temporal rhythm in working memory, and that this is not dependent on overt motor actions. This will support the notion that rhythmic interval timing is a higher cognitive function not tied to particular motor actions, which is shared among primates.

We developed a rhythm task in which subjects had to observe a visual stimulus that periodically changed its location along a circular perimeter. After this *presentation* period, the stimulus disappeared and subjects had to internally follow its location as a function of elapsed time. Importantly, at a random time during this *continuation* phase, subjects were asked to indicate the estimated position of the stimulus (*Go-time*). Thus, this task generated a visuospatial rhythm defined by the time interval between location changes (Doherty et al., 2005), much like the rhythm defined by the motion of a discretely moving second hand in a clock. To correctly estimate the stimulus position subjects must first adjust their internal chronometers to the pace of the visual stimulus and then use that internal rhythm to predict the position during the continuation phase. Since we know that the variability of the timing estimates increases with elapsed time we expect the probability of correct responses to decline as a function of time.

Whether subjects time single intervals independently or they estimate total elapsed time is an important open question that we address in human subjects by analyzing the pattern of errors and also by fitting *continuous time* and a *reset time* models.

An important question in timing research is whether intervals of different lengths are timed by a single mechanism or whether different intervals use distinct chronometers. There is evidence that the standard Weber fraction is not constant for intervals larger than approximately 1.2 s (Hinton and Rao, 2004; Bizo et al., 2006; Lewis and Miall, 2009; Grondin, 2012, 2014; Allman et al., 2014), and this could be a sign that different clocks or timing processes are used to time intervals of different durations (Bangert et al., 2011; Rammsayer and Troche, 2014). We approach this issue by calculating the traditional Weber fraction for intervals of different duration, and also by fitting a model of the generalized Weber fraction (Equation 1). The results show that the Weber fraction diminishes as a function not only of total elapsed time, but also as a function of the interval length subdividing that total time (Grondin et al., 1999). Our results demonstrate that the generalized Weber law provides a satisfactory description of behavioral patterns such as the proportion of correct responses, the increase in variability as a function of time, and the systematic pattern of timing errors. The evidence suggests that short (0.5 s), medium (0.75 s), and long intervals (1.0 s) seem to be timed by mechanisms with increasingly large time-independent variance.

## Methods

### Behavioral tasks

**Figure 1:**
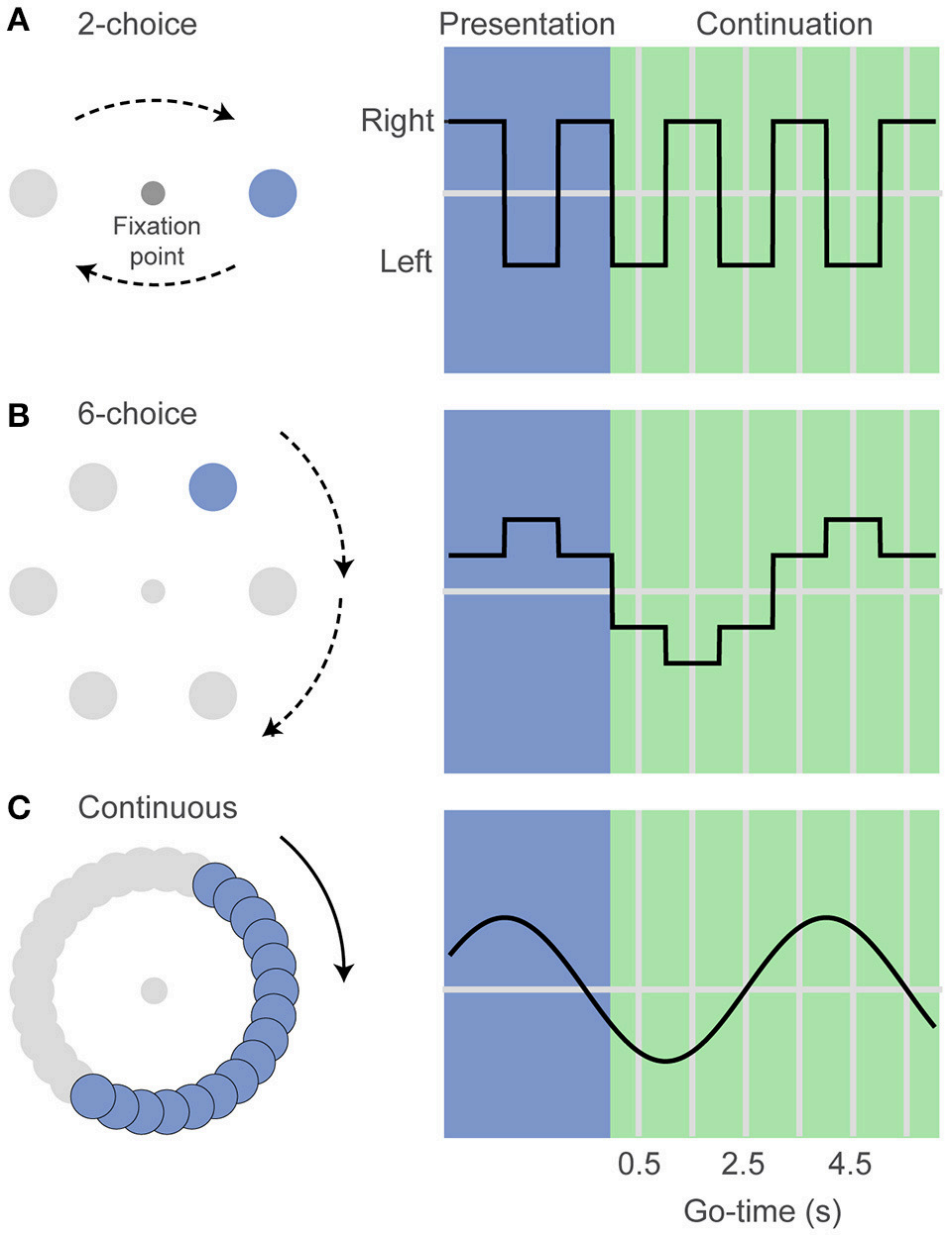
**The visuospatial rhythm task. (A)** In the *2-choice* task a visual stimulus (colored disk) alternates left and right of a central fixation point. After three visible intervals (*presentation* phase) the disk disappears and subjects must internally track its location as a function of elapsed time (*continuation* phase). The fixation point can disappear at the midpoint of any pseudo-randomly selected *continuation* interval (*Go-time*), instructing the subjects to indicate the estimated disk location (left or right). **(B)** In the *6-choice* task the disk moves sequentially to six marked locations along a circular path. Taking into account the direction of rotation, this version of the task allows estimating whether the subjects' responses are ahead of or behind the correct target location. **(C)** In the *continuous* version the disk moves smoothly along a gray path at a velocity that matches the position of the disk on the *6-choice* task. Angle units were transformed to time units for the analyses described in the text.

In our visuospatial rhythm task the human subjects were asked to maintain their eyes in a fixed position (fixation) and to keep a mouse cursor at the center of a computer monitor while attending a peripheral disk that periodically changed location (Figure 1). After the presentation of 3 filled intervals (*presentation* phase), the disk disappeared and subjects had to covertly predict its position as a function of elapsed time (*continuation* phase). After 1–6 *continuation* intervals (uniform distribution, pseudo-randomly selected) the fixation point disappeared (*Go-time*), instructing the subjects to move the cursor and click over estimated position of the disk at the *Go-time*. It is important to note that the rhythm stops at *Go-time* and subjects can calmly click over the estimated position. In other words, it is not an interception task in which reaction time and hand movement should be taken into account when executing the behavioral response. The interval duration was chosen pseudo-randomly on each trial (0.50, 0.75, or 1 s for monkey and *8-choice* datasets; 0.50 or 1 s for the rest of the datasets). Instead of using a mouse, monkeys were trained to maintain their right hand at the center of a touchscreen and at the *Go-time*, to perform a reach movement to touch the estimated location of the disk. They were rewarded with a drop of water on correct responses. An infrared camera (200 Hz, Applied Science Laboratories) was used to monitor eye position within 1.5° around the fixation point (Figure 1).

Monkeys were first trained in a 6-choice version of the task but then we decided to simplify it to a 2-choice task that is more suitable for the acquisition of neurophysiological data that we plan to carry after the behavioral tests presented in this report. (Figure 1). In addition to the 2-choice task, human subjects performed a *6-choice*, an *8-choice*, and also a *continuous* version of the task. The *6-choice* and *8-choice* versions of the task were included in the human experiments to accurately estimate how the variance of the behavioral responses changes as function of elapsed time. The use of 6 or 8 targets make it possible to measure whether responses are ahead of or behind the true stimulus position. This is not possible in the *2-choice* task because there is only one correct and only one incorrect target.

In the *continuous* version of the task, the disk moved smoothly along a gray path. The disk moved at the same speeds as those in the *6-choice* task. A response was defined as correct if the mouse click was within 30° of the correct position (this divides the gray circular path into six regions, analogous to the *6-choice* task). We developed the *continuous* task as a control experiment in which timing is required to estimate the position of an invisible target (O'Reilly et al., 2008), but it does not depend on the rhythm imposed by the repetition of isochronous intervals.

To correctly predict the stimulus position subjects must rely on an internal chronometer whose variability increases with elapsed time, as described by the generalized Weber's law. Thus, the *Go-time* is a key experimental variable determining how well the subjects can estimate the disk location. Short *Go-time*s will likely result in correct responses, while at long *Go-time*s subjects are more likely to miss the correct disk location (they can get ahead or behind the true location). Note that the spatial location of the stimulus (angle) and the spatial location of the behavioral responses (angle) were expressed in time units (seconds).

We describe behavioral performance with four variables, and we plot these as a function of *Go-time* (**Figure 4**): (1) The probability of a correct response *p(correct)*, indicating the proportion of trials in which subjects correctly estimated the position of the disk; (2) the standard deviation (*Std*) of the responses, expressed in time units; (3) the traditional Weber fraction, defined as the standard deviation (*Std*) divided by the mean generated time (mean spatial location of the behavioral responses, converted to time units); and (4) the constant error, or bias, defined as the difference between the true and the estimated position of the disk, expressed in time units. It must be noted that the constant error can only be estimated in the *6-choice, 8-choice*, and the *continuous* versions of the task. The *2-choice* version of the task allows recording correct and incorrect responses, but precludes determining whether an incorrect response was ahead of or behind the true stimulus position. The columns of **Figure 4** show these four behavioral parameters for each dataset, grouped by interval duration, and plotted as a function of *Go-time*. The *Go-cue* (disappearance of the eye fixation point in humans or disappearance of hand fixation point in monkeys) occurred at the middle of 1–6 continuation intervals (pseudo-randomly selected; 1–4 continuation intervals in monkeys). Thus, *Go-times* were 0.5, 1.5, 2.5, 3.5, 4.5, 5.5, for the 1 s interval and 0.25, 0.75, 1.25, 1.75, 2.25, 2.75 for the 0.5 s interval. For monkeys, the first four of those *Go-times* were used, and an additional interval of 0.75 s was also tested (*Go-times* 0.38, 1.13, 1.88, 2.63 s).

### Participants, apparatus, and training

Thirteen human subjects were tested in this study and were paid for their participation (8 females, median age 25, Std 4.1). They were right-handed, had normal or corrected-to-normal vision, and were naive about the purpose of the experiment. All subjects reported no systematic musical training for more than a year. Each subject volunteered and gave informed consent for this study, which complied with the Declaration of Helsinki and was approved by the National University of Mexico Institutional Review Board. In addition to a minimum monetary compensation, human subjects were also compensated for every correct trial (feedback was provided on each trial by flashing the correct target position). Two male monkeys (*Macaca mulatta*, 5–7 kg, ages 5, and 6) were used. Animal experimental procedures were approved by the National University of Mexico Institutional Animal Care and Use Committee and conformed to the principles outlined in the Guide for Care and Use of Laboratory Animals (NIH, publication number 85-23, revised 1985). Human subjects were seated comfortably on a chair facing a computer monitor (LCD screen, 60 Hz refresh rate, model S27C350H) in a quiet room. Stimuli were generated and data were collected with custom software written in Matlab and the Psychophysics Toolbox (Brainard, 1997). Subjects came to the lab on separate days to perform each task type (*2-choice, 6-choice, 8-choice, continuous*). The order of the task type was counterbalanced between subjects. In each session subjects performed 48 training trails followed by a 15 min rest period, and then 288 test trials (6 Go-times, 2 interval durations, 24 repetitions) with 15 min rest periods every 98 trials. Monkeys spent ~4 months progressively learning the task structure, and another ~6 months for their performance to reach asymptotic levels. To make sure monkeys learned to estimate a rhythm (*presentation* phase) and then being able to use that rhythm to predict the stimulus position as a function of the elapsed time (*continuation* phase), we first trained them in a version of the 6-choice task in which interval length was chosen from a continuous distribution (300–1200 ms, uniform distribution) and the number of *presentation* intervals was variable (1–4, uniform distribution). This variation in initial conditions minimized the possibility of monkeys learning a simple association between elapsed time and a fixed stimulus position on the screen. We then moved to the *2-choice* task that we present here and that will be used in physiological recordings in the future. The 2-choice version of the task is better suited for the acquisition and analysis of neuronal data because it has fewer conditions and variables. For example, it has only two possible starting and end locations of the stimulus. The behavioral decision is thus binary, allowing us to record many repetitions of the same type of trials and the underlying neurophysiological data. On each training day monkeys performed 3–6 runs with approximately 130 trials per run. The data analyzed here was obtained from 358 sessions in a ~4 month period following training (109 sessions monkey I; 249 sessions monkey M; 47,235 total trials).

### Fitting the generalized Weber law

**Figure 2:**
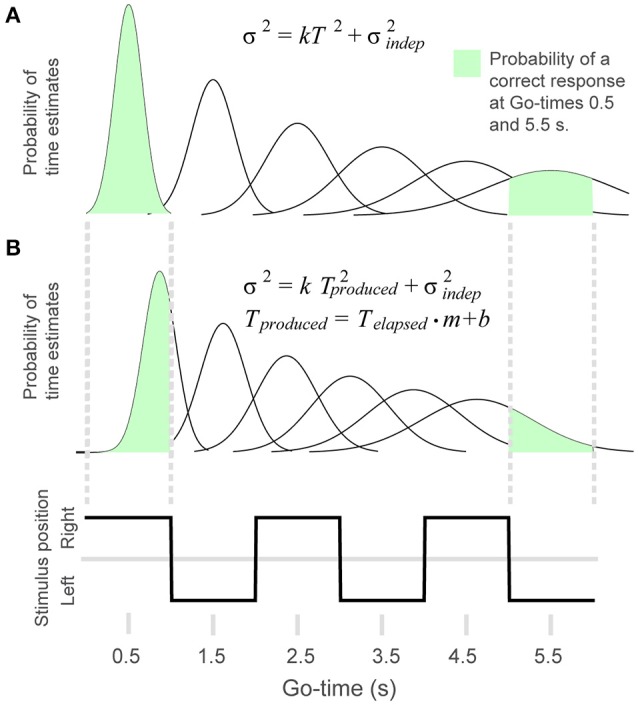
**To model behavioral performance we adapted the generalized Weber law to the rhythm task. (A)** The Gaussian distributions illustrate the time-dependent increase in variability of the timing estimates. The probability of a correct response was calculated as the area of the Gaussian curve comprised within the interval defined by a given *Go-time*. The probability of a correct response for *Go-time*s 0.5 and 5.5 s is illustrated in green. **(B)** In the 4-parameter equation, produced time is modified by multiplicative and additive factors. This allows the model to capture systematic errors like shortening or lengthening of elapsed time. The figure illustrates the distributions resulting from a positive displacement and a shortening of time estimates.

To test the extent to which behavioral performance conformed to the scalar property of timing, we adapted the generalized Weber law to the discrete time intervals that define our rhythm task (Figures 2A,B). We generated a model in which the probability of a correct response *p(correct)* was defined as the area under a Gaussian distribution that is comprised within the limits of the time interval corresponding to a given *Go-time* (this distribution represents the variability of the internal time estimates). For example, Figure 2A shows that the area comprised within the first *continuation* interval (*Go-time* = 0.5 s) is close to 1, whereas the area comprised within the sixth memory interval approximates 0.5 (*Go-time* = 5.5 s). In this manner, as described by the generalized Weber law (Equation 1), the time-dependent increase in variability results in a reduced proportion of correct responses as a function of *Go-time*, and the steepness of this decrease is modulated by the *k* parameter of Equation 1.

In its traditional form, the Weber fraction determines the slope with which the standard deviation of time estimates grows as a function of elapsed time: σ = *k*·*T*; where *k* is the Weber fraction, σ stands for standard deviation and *T* is elapsed time. However, it has been found that the addition of a time-independent noise constant better describes how σ grows as a function of time: σ = *k*·*T* + σ_*indep*_; in which σ_*indep*_ represents this time-independent source of variability. The addition of this constant results in the traditional Weber fraction (σ /mean) not being constant as a function of elapsed time: it is higher at short times and it decreases as time elapses. This is because at short times (*T* is small) the total variability is dominated by σ_*indep*_, and as time elapses the total variability is mainly due to the *k*·*T* product. Thus, at longer elapsed times the term *k* in Equation 1 approximates the traditional Weber fraction in which the variability is accounted by *k*·*T*. When variability is expressed as variance and time is also squared, the resulting equation for the generalized Weber fraction is Equation (1).

In addition to fitting *p*(*correct*), our model also fit the standard deviation (*Std*) of the behavioral responses. However, it is not possible to directly fit Equation 1 to our data because of the discrete nature of the behavioral responses (2-, 6-, 8-choice), i. e., Equation (1) varies continuously whereas the subjects' responses vary within a finite number of options. Thus, the model calculates Std from the expected proportion of responses distributed across the discrete time intervals. In the case of the *2-choice* task, for example, the discrete nature of the responses causes the standard deviation to saturate at long elapsed times, when behavior is at random chance and the behavioral responses are distributed equally between the two choices (**Figure 4**, second column). Thus, the 0.5 s saturating value is the expected standard deviation of a random variable taking the values 0 and 1 s (as in the behavioral responses corresponding to correct and incorrect responses in the 1 s time interval trials).

The generalized Weber law describes how variance changes as a function of time. However, it cannot account for systematic trends in the constant error that is, it cannot capture whether a subject's estimate of time is ahead of or behind true elapsed time. For our model to capture systematic differences between real and estimated time (constant error, **Figure 4**, rightmost column) we made use of two additional parameters (*m, b*):

(2) $$\begin{array}{r} \sigma^{2}=k\cdot T_{produced}^{2}+\sigma_{indep}^{2}\\ T_{produced}=T_{elapsed}\cdot m+b \end{array}$$

These parameters allowed our model to take into account biases such as a constant time displacement (*b*), and the shortening or lengthening of produced time (*m*) (Figure 2B). Equation 2 was used for the fits shown in Figures **4C,D**. However, when comparing parameters *k* and $\sigma_{indep}^{2}$ across tasks we used the two-parameter generalized Weber's model (Equation 1, this is because the 2-choice tasks do not allow to calculate the constant error).

As was done by Buonomano and colleagues (Laje et al., 2011), we tested a *reset* version of the generalized Weber law in which, instead of variance increasing in proportion with total time squared (term *k*·*T*^2^, Equation 1), it increased with the sum of the squares of each interval duration:

(3) $$\begin{array}{r} \sigma^{2}=k\cdot \left(T_{1}^{2}+T_{2}^{2}+\ldots +T_{Go-time}^{2}\right)+\sigma_{indep}^{2} \end{array}$$

**Figure 3:**
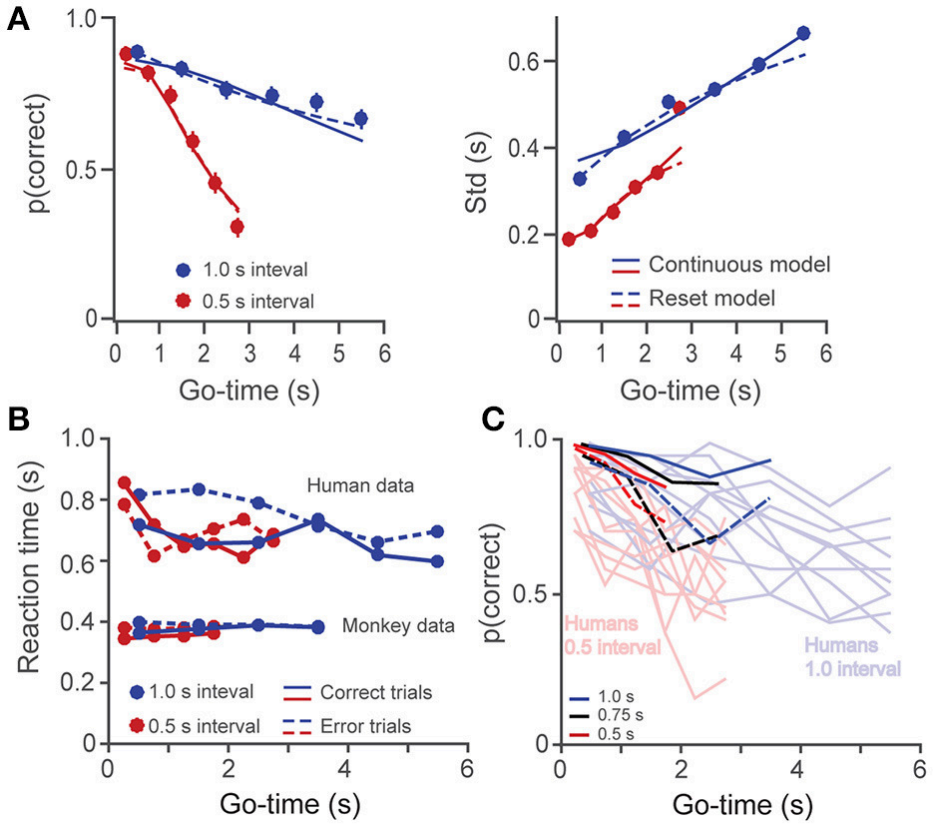
**(A)** The probability of a correct response (left panel) and the standard deviation (right panel) are plotted as a function of *Go-time*, separately for the short and long intervals (0.5 and 1 s, human data on the *6-choice* task). Solid and broken lines depict model fittings of a *continuous* (Equation 1) and a *reset* (Equation 3) model of timing. Both models provided similarly good fits. **(B)** Reaction times as a function of *Go-time*. Humans had significantly higher reaction times which tended to decrease with *Go-time*. **(C)** Single subject data for monkeys (*n* = 2) and humans (*n* = 10) in the 2-choice task. The probability of correct responses *p(correct)* is plotted as a function of *Go-time*. Light colors are used for humans and dark ones for monkeys. Broken dark lines as used for monkey 2 data.

Thus, in the *reset* version of the model, variance increases linearly rather than quadratically with time. By plotting Std as a function of Go-time, this trend can be observed as a saturating effect at large *Go-time*s (Figure 3A, right panel). Whether subjects time individual intervals separately, or they time total elapsed time is an important question in timing research (Hinton and Rao, 2004; Hinton et al., 2004; Laje et al., 2011; Narkiewicz et al., 2015). We found that the *continuous* (Equation 1) and *reset* (Equation 3) models provided statistically similar fits to our data (*p* = 0.13, paired *t*-test on the Fisher-transformed correlation coefficients between behavioral data and model estimates, *t*_(24)_ = −1.6; Laje et al., 2011; Figure 3A). For simplicity, our model used Equations 1, 2 to fit the behavioral data.

Fitting was performed with the function *fmincon* in Matlab R2014b by minimizing the error between estimates from the model and the behavioral results, simultaneously for parameters *p(correct)*, Std, and constant error (one fit for each interval duration). Because of the difference in scale and measurement units [probability in *p(correct)*, seconds in Std, and constant error], these quantities were standardized to values between 0 and 1 before calculating the total fitting error.

## Results

**Figure 4:**
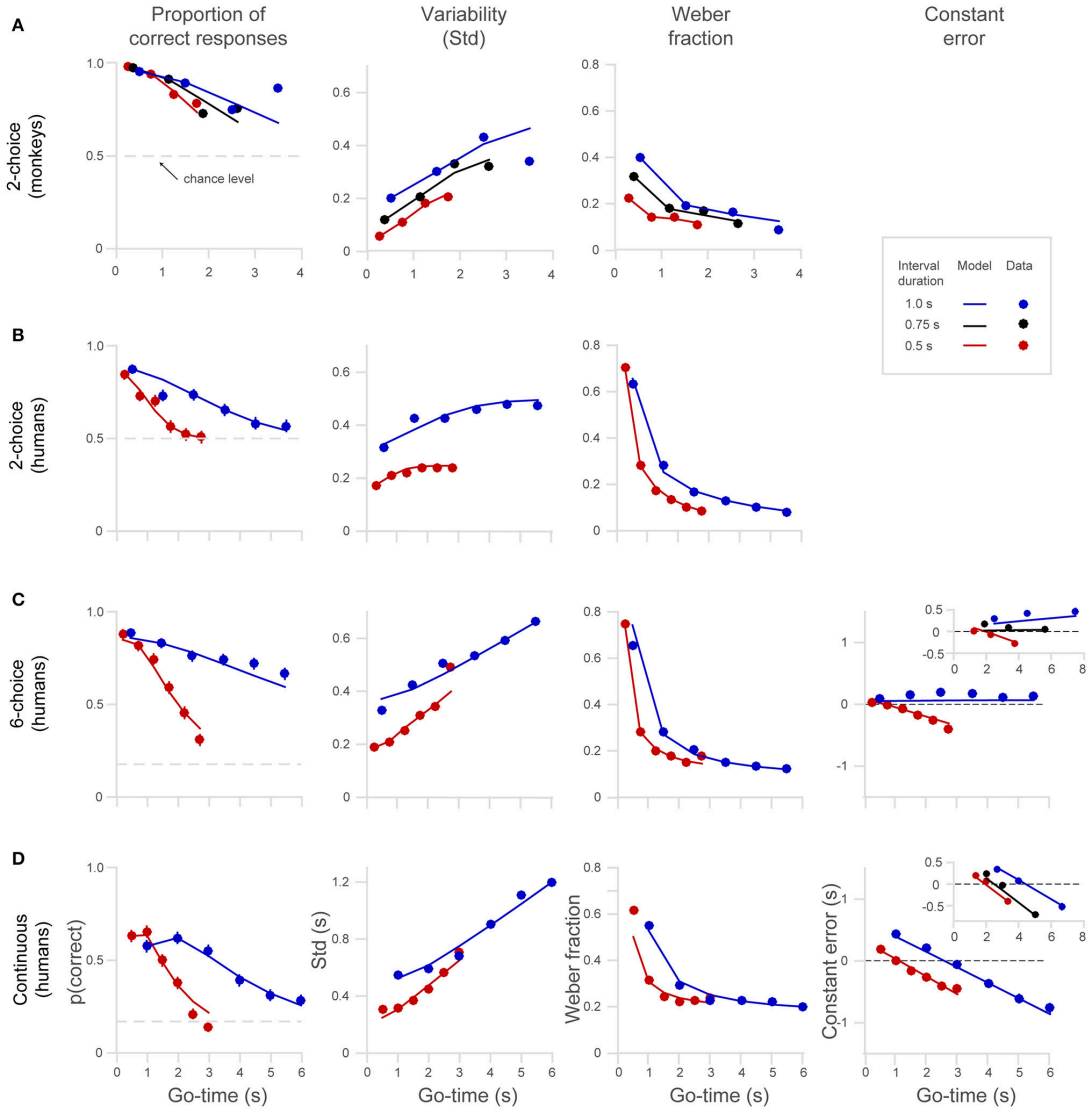
**Behavioral performance of humans and monkeys in the ***2-choice***, ***6-choice*** and ***continuous*** tasks. (A)** Monkeys' performance on the *2-choice* task. Note that they performed up to four *continuation* intervals. **(B)** Human performance on the *2-choice* task. (**C**) Human performance on the *6-choice* task. **(D)** Human performance on the *continuous* task. The columns from left to right show the probability of a correct response *p(correct)*, standard deviation *Std*, Weber fraction, and constant error (note that constant error cannot be calculated in the *2-choice* tasks, see Section Methods). Continuous lines show model fits (Equation 1) to the different interval lengths (red 0.5 s, black.0.75 s, blue 1.0 s). The insets in the fourth column show the constant error in an *8-choice* task and in a *continuous* task that included a 0.75 s interval. All panels share the axes notation of **(D)**.

Humans and monkeys learned to perform the timing tasks, and their behavior showed consistent patterns. We show single subject data for the 2-choice task in Figure 3C and mean data for the different datasets in Figure 4. The proportion of correct responses (Figure 4, first column) decreased as a function of *Go-time*, a trend well captured by our model of the generalized Weber law (continuous lines). Monkeys' performance (Figure 4A) was better than that of humans (Figure 4B) as can be readily appreciated by the larger proportion of correct responses, the lower variability (Std), and the lower Weber fraction. In humans, the proportion of correct responses approached random performance around the 5–6^th^ intervals, and the standard deviation saturated at 0.5 and 0.25, the maximum possible values for the 1 and 0.5 s intervals on the *2-choice* task (Figure 4B, see Section Methods).

In addition to a better performance, monkeys also showed significantly faster reaction times to the *Go-cue* (Figure 3B, *p* < 0.01, two-sample *t*-test on the pooled data for humans against the pooled data for monkeys, i.e., all *Go-times*, correct and incorrect responses; *t*_(38)_ = −17.9). It is likely that increased performance and faster reaction times are a consequence of the longer training the monkeys received (Methods and Discussion). Human subjects showed a trend of diminishing reaction times as a function of *Go-time* (linear regression, slope = −20 ms/s, *p* < 0.05), which could reflect the anticipation of trial termination (increasing hazard rate).

Compared to the *6-choice* task, the proportion of correct responses in the *continuous* task was significantly lower (Figures 4C,D, to formalize this observation we performed a paired *t*-test comparing each *p(correct)* across tasks for each *Go-time* and each interval length *t*-test, *t*_(11)_ = 11.2, *p* = 2.4e-07.). As described in Section Methods the region defining a correct response in the *continuous* task was a window of ±30° around the correct location, comprising a 6^th^ of the circle, just as in the *6-choice* task. However, it is likely that the larger variability and the resulting lower proportion of correct responses observed in the *continuous* task is explained by the absence of six defined choices. With six defined choices, there is less uncertainty about the correct target position.

As can be observed on the panels of the first column of Figure 4, the decreased proportion of correct responses is more pronounced for the short interval (0.5 s, red dots and lines), and this is observed in all versions of the task (*2-, 6-choice*, and continuous). To formalize the observation that *p(correct)* decreases more rapidly for short intervals (0.5 s) than for long ones (1.0 s) we compared *p(correct)* at similar intermediate *Go-times* for each dataset (one *p(correct)* for each interval, i.e., comparison of two proportions for each dataset). We set *p* < 0.01 and then we corrected for multiple comparisons (Bonferroni correction, new significant *p* < 0.0025). That *p(correct)* decreases more rapidly for fast intervals is an expected trend because the temporal window for a correct response is narrower for short intervals. That is, even if timing variability at a given elapsed time is equal for short and long intervals, a reduction in the probability of correct response is expected for narrower time intervals.

We observed, as had other studies before, that the Weber fraction is not constant but declines exponentially as a function of time (Figure 4, third column; Laje et al., 2011). This trend is explained by the presence of time-independent variability (y-intercept on the Std graphs, term $\sigma_{indep}^{2}$ of the model). This basal variability has a large influence at short elapsed times. At longer elapsed times the y-intercept has less impact on the ratio *Std/mean* that defines Weber fraction. The fact that the generalized Weber law satisfactorily fits the behavioral data is strong evidence supporting the presence of time-independent variance in the timing mechanism (Figure 4, third column).

### Constant errors and its relation to timing strategy

In addition to the proportion of correct responses *p(correct)*, variability (*Std*), and Weber fraction, the *6-choice* and *continuous* tasks allowed us to estimate the constant error, i.e., the difference between estimated and true elapsed time. This is easily computed by taking into account the direction of disk rotation (clockwise or counterclockwise) and then calculating the difference between the true disk position and the subject's estimated disk position. This angle difference is then expressed in time units. We observed a marked difference in the pattern of errors between the *6-choice* and the *continuous* versions of the task, and this difference can be useful to determine whether subjects are timing each individual interval or total elapsed time.

When the target jumps fast across the six choices (0.5 s interval, red dots, Figure 4C), the pattern of negative errors indicates that subjects increasingly lag behind the real target position, thus signifying that the subject's internal chronometer was running slower than the intended pace (Figure 4C, last column, red line and dots). Showing the opposite trend, subjects tended to get ahead of a slowly jumping target (1 s interval, blue dots, Figure 4C), indicating that their internal chronometer was running faster than the intended 1 s intervals. As can be observed in the insert, the same pattern of errors was observed in an *8-choice* task in which three interval durations were tested (0.5, 0.75, and 1 s). Importantly, the insert shows that the behavioral responses for the middle interval duration (0.75 s) were unbiased, suggesting that the subjects' internal chronometer tends to pace at the rate that is the mean of the distribution of interval durations (Jazayeri and Shadlen, 2010, 2015). We performed a one-way analysis of covariance (ANCOVA) on the mean constant errors and found that slopes are significantly affected by the “interval duration” factor. This analysis also revealed that the slope for the 1 s interval is significantly positive, (*p* < 0.01, *t* = 7.7, d.f. = 3; inset on Figure 4C, blue dots), the slope of the 0.75 s interval is not significantly different from zero [*p* = 0.61, *t*_(3)_ = 0.6] and finally, that the slope of the 0.5 interval is significantly negative [*p* < 0.01, *t*_(3)_ = −6].

Compared to *6-choice*, the *continuous* task shows a different pattern of errors as can be readily appreciated in the last column of Figure 4D. Instead of a bias that progressively accumulates with a positive slope for long intervals and a negative slope for short ones, what it is observed is that all interval durations generate constant errors with negative slopes. Moreover, for all interval durations, short elapsed times (*Go-time*) generate positive errors while long elapsed times result in negative errors. The same trend can be observed in the insert depicting a *continuous* experiment in which three disk speeds were used (matching the position of the disk in the *6-choice* task at the 0.5 and 1.0 s intervals, with an additional interval of 0.75 s).

**Figure 5:**
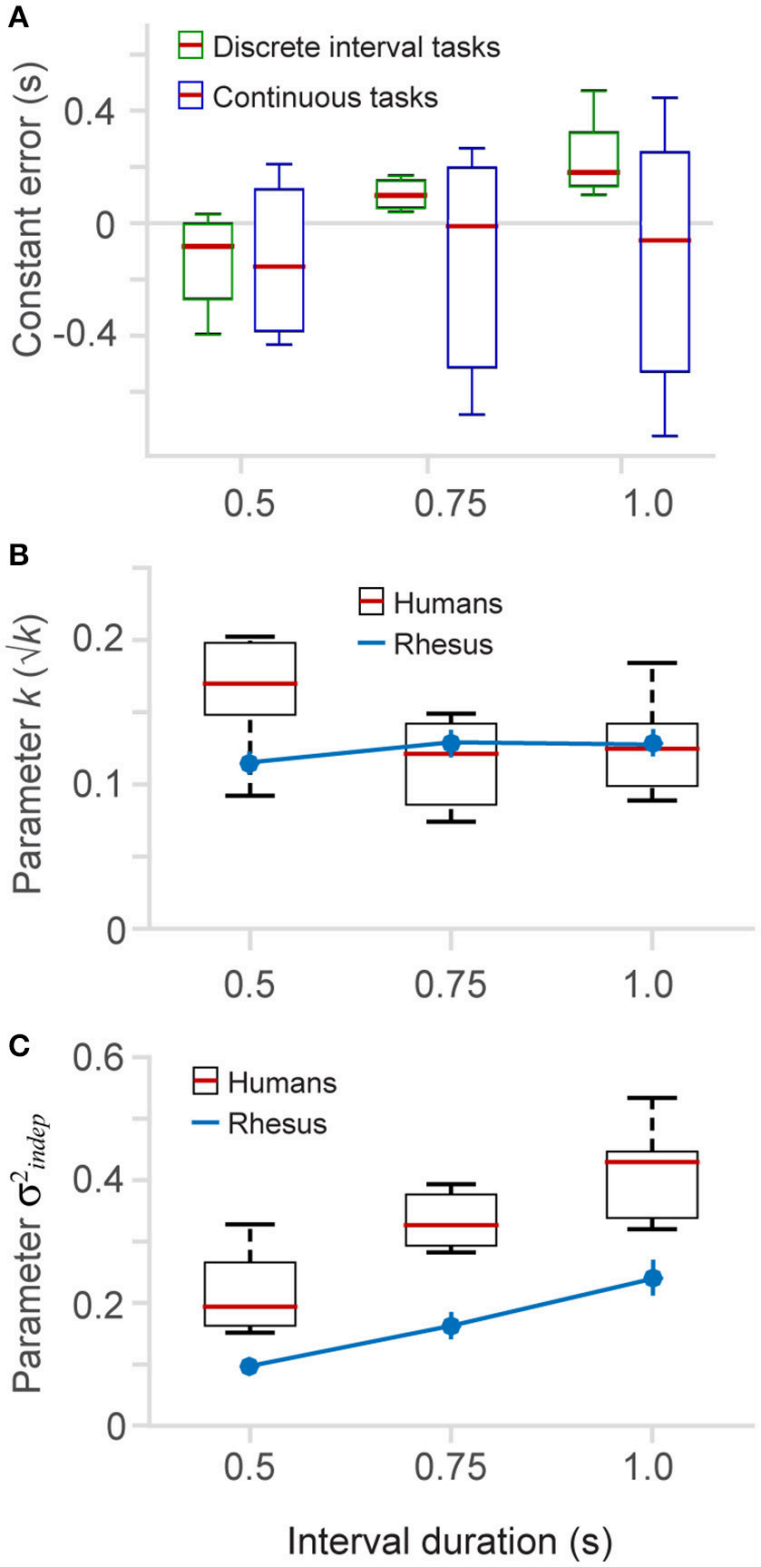
**Constant error and fitted parameters. (A)** The constant error (difference between produced and true elapsed time) is plotted for the different time intervals (0.5, 0.75, and 1.0 s), separately for the discrete (6 and *8-choice*) and *continuous* versions of the task. **(B)** The fitted parameter *k* (Equation 1) as a function of interval duration (boxplots human data, *n* = 6; blue lines monkey data, *n* = 2) (2-choice, 6-choice, 8-choice (insert in Figure 4C), continuous with three interval durations (insert in Figure 4D), and also from a dataset of the continuous task that is not show in results). **(C)** Parameter $\sigma_{indep}^{2}$ (Equation 1) as a function of interval duration.

Constant errors are plotted as a function of interval length in Figure 5A, separately for the discrete and *continuous* tasks. It can be seen that the constant errors in the discrete tasks (pooled 6- and *8-choice;* averaged across *Go-times*) change with a positive slope as interval length increases, whereas in the *continuous* tasks they span both positive and negative values for all interval durations. We conducted a linear regression on each dataset (*continuous* and discrete) and found that constant errors on the discrete task have a significantly positive slope (0.74, [0.47 1.0] 95% C.I., d.f. = 19), and a significantly negative y-intercept (0.4958, [0.7039–0.2877] 95% C.I., d.f. = 19), i.e., they go from negative to positive values as interval duration increases. Conversely, the regression on the continuous task shows that the slope and intercept are not statistically different from zero, i. e., they are scattered around zero for the three interval durations (slope = −0.02, [−0.74 0.71] 95% C.I., d.f. = 19; intercept = −0.12, [−0.68, 0.45] 95% C.I., d.f. = 19).

The error patterns differ between the *continuous* and *discrete* tasks, suggesting that in the discrete *8-choice*, and *6-choice* tasks subjects are timing individual intervals and that their estimates are biased toward a mean interval. Conversely, in the *continuous* task the pattern of errors indicates that subjects were timing the total duration of the *continuation* phase, and their time estimates are biased toward the mean total duration (Jazayeri and Shadlen, 2010; Acerbi et al., 2012). Our finding that the continuous and discrete tasks exhibit different error patterns is important because it allows us to determine whether subjects are timing individual intervals or total elapsed time (see Section Discussion).

### Time dependent and time-independent variance

The brain might use a single chronometer to time a range of durations or, conversely, make use of different chronometers for different behaviorally relevant intervals. This question can be approached by comparing the classical Weber fraction in long- and short-interval trials, as illustrated in Figure 4 (third column), and also by comparing the coefficients *k* and $\sigma_{indep}^{2}$ (Equation 2) resulting from fitting the model to the behavioral data, separately for each time interval. If a single chronometer underlies timing of short and long intervals, we would expect similar Weber fractions and similar *k* and $\sigma_{indep}^{2}$ values for the different interval durations. Significant differences in these parameters would lend support to the notion that multiple chronometers could be used to time different intervals.

As described by Weber's law, the standard deviation of the timing estimates linearly increases with elapsed time. The human data on the *6-choice* and *continuous* tasks indicate that this increase in variability has different slope and intercept values for long- and short-interval trials (Figures 4C,D, Std graphs). Short-interval trials (0.5 s) have smaller variability but a larger slope, while long-interval trials (1 s) show a larger variability that grows at a lower rate (variability patterns on the *2-choice* version of the task are no informative because they have an upper limit at long elapsed times, and this limit is different for long and short intervals, see Section Methods). We found that, the traditional Weber fraction decreases as a function of elapsed time (Laje et al., 2011), and additionally, that short-interval trials show lower Weber fractions for elapsed times up to 3 s.

To quantitavely assess whether variability differs across interval durations (0.5, 0.75, and 1 s) we fit our datasets with the two parameter model (Equation 1) to estimate the *k* and $\sigma_{indep}^{2}$ parameters. Figures 5B,C plot the fitted parameters as a function of interval duration. In humans, we observed a tendency of *k* to be larger for the short interval (Figure 5B). However, this tendency was not present in the monkey data, indicating either a difference between species or possibly an effect of training on the *k* parameter. We speculate that human subjects showed a larger *k* parameter because they performed fewer trials of the timing task (as presented next, this is also the case for the $\sigma_{indep}^{2}$ parameter, an observation also made by Laje et al., 2011).

Our results show a positive correlation between the $\sigma_{indep}^{2}$ parameter and interval duration (Figure 5C). Longer time intervals show larger $\sigma_{indep}^{2}$, and this trend is observed in humans as well as in monkeys. Monkeys, however, have lower $\sigma_{indep}^{2}$ values, probably due to an effect of additional training and the total number of trials they performed (see Section Methods). We tested this correlation by a linear regression and found that for panel 5B the slopes for monkeys and humans are not statistically different from zero, meaning that there is no influence of the interval length on the *k* parameter slope for human data: −0.07, [−0.17 0.02] 95% C.I., d.f. = 13; slope for monkey data: 0.02, [−0.20 0.25], 95% C.I., d.f. = 1. For Figure 5C we found that both linear regressions have statistically significant positive slopes (slope for human data: 0.4, [0.23 0.57] 95% C.I., d.f. = 13; slope for monkey data: 0.28, [0.12 0.44], 95% C.I., d.f. = 1), meaning that the basal standard deviation (parameter $\sigma_{indep}^{2}$) increases as a function of interval duration.

## Discussion

In summary, the main novel observations of the present study are that (1) monkeys were as capable as humans to follow visuo-spatial rhythms with different tempos, and they were able to internally maintain those rhythms without overt movements; (2) both species showed an increase in temporal variability that followed the generalized Weber law, where the time-independent variability changed as a function of the tempo (interval length); and (3) the pattern of constant errors across tempos indicated that human subjects were resetting their clock each interval instead of measuring continuous elapsed time.

### Monkeys and humans can internally maintain a temporal rhythm

Our experiments show that monkeys and humans are able to *perceive* visuo-spatial rhythms of different paces, and they can internally maintain those rhythms without overt movements. This important finding indicates that rhythm perception and maintenance is a higher cognitive function that we share with other primates and that it does not depend on the execution of motor commands.

The pattern of constant errors (Figures 4C,D, last column) calculated from the human data suggests that subjects were timing individual intervals in the discrete task, but total duration in the *continuous* task. Additionally, timing errors in the *6-* and *8-choice* tasks show that subjects were lagging behind fast rhythms and getting ahead of slow ones (although the errors in 1s interval of the 6-choice task do not increase linearly they are all positive. The increasing trend is better appreciated in the insert of Figure 4C). The fact that a rhythm of intermediate pace generated no bias supports the notion that the timing mechanism calibrates itself to the distribution of interval durations it has to measure, as has been shown by previous research (Jones and McAuley, 2005; Jazayeri and Shadlen, 2010, 2015; Acerbi et al., 2012). Conversely, timing total elapsed time generates a pattern of errors that are positive for short elapsed times and negative for long elapsed times. This suggests that subjects' time estimates were biased toward the mean total duration. We propose that the different patterns of constant errors are a reliable signature that could help to distinguish whether subjects are timing individual intervals or total elapsed time.

The tendency to produce intervals that are closer to the mean is a well-established observation often named the “central-tendency” effect or Vierordt's law (Roy and Christenfeld, 2008; Bangert et al., 2011; Shi et al., 2013). Our results show that in keeping rhythms of different paces the central tendency effect is observed as a bias toward the mean frequency of the rhythms instead of toward the mean total duration. Incorporating prior information such as the mean value of a range of intervals is a mechanism that helps to reduce the effect of noise in time estimation and production, and in our case, rhythm maintenance.

We did not test our monkeys on the continuous task so whether they show the same pattern of errors as human remains an open question. However, it is important to consider that monkeys and humans showed the same patterns of behavioral responses in the 2-choice task, and also the same model satisfactorily accounted for the behavior of human and monkeys.

The question whether subjects time individual intervals or total duration has been addressed before in humans (Hinton and Rao, 2004; Hinton et al., 2004; Laje et al., 2011; Narkiewicz et al., 2015). Buonomano and colleagues used a spatiotemporal task in which subjects had to perform a series of button presses with an elaborated spatial and temporal structure. They found that, although subjects were generating a series of individual intervals, a *continuous* time model was a better fit to their behavioral results. On the contrary, our data from the *6-choice* task suggest that subjects were resetting their clocks after each individual time interval. We believe these seemingly contradictory results arise from the different experimental designs. In our rhythm task, subjects could be asked to indicate the position of the target at any given interval (*Go-time*), so they were prepared to generate a behavioral response for each interval. If the Go cue didn't arrive by the middle of an interval they had to start timing the next interval and so on. In contrast, on the rhythm task of Buonomano and colleagues subjects had to perform a complete series of intervals for each trial, and this might have compelled them to time total elapsed time. We think that variable *Go-time*s, that is, the possibility of terminating the trial at any interval, prompted the subjects to time each interval independently.

It might seem contradictory that the pattern seen in the constant errors suggests that subjects were timing individual intervals whereas the model we fit was based on variance growing with total elapsed time (Equations 1, 2, human data). However, we must note that the difference between a *reset* and a *continuous* model, from the point of view of how variability grows, is a difference in the shape of the curve of Std vs. time (Figure 3A). The *reset* model predicts that *Std* grows sub-linearly while the *continuous* model predicts a linear increase. We found that, with our current data, these two models could not be distinguished. Our results showed, however, that *continuous* and a *reset* mode of timing could be discerned from the pattern of constant errors (Figures 4C,D, last column).

### Basal variance depends on interval length

Monkeys and humans showed performance parameters well captured by the generalized Weber law. Monkeys, however, showed less timing variability and a higher proportion of correct responses. We speculate that this superior performance is due to the longer training the monkeys received (the monkey dataset was collected after 4–6 months of training). It is likely that increased performance and faster reaction times are a consequence of the longer training the monkeys received. However, it is also possible that differences in reward value and motor planning also contribute to these differences (humans used a mouse cursor while monkeys directly touched the screen to communicate their choices). Previous studies in humans have shown that the Weber fraction quickly decreases after just a few practice sessions (Laje et al., 2011). We speculate that due to their extensive training the Weber fraction of our monkey subjects was at its asymptotic value, but this might not have been the case of our human subjects who performed only one practice session. We expect that with enough training, human subjects could have performed the rhythm task as accurately as the monkey subjects. Our model fittings revealed that humans and monkeys had similar *k*-values (Equations 1, 2, Figure 5B), and that the lower variability of the monkeys' time estimates was due mainly to a lower time-independent variance (Figure 5C, blue line).

The term $\sigma_{indep}^{2}$ showed a tendency to increase as a function of interval duration in both species, indicating that different time intervals have different amounts of time-independent noise. This observation suggests that different chronometers or time mechanisms could time different interval durations. We favor the view that training in timing tasks induces the formation of multiple time templates that match the range and distribution shape of the behaviorally relevant time intervals. Indeed, previous psychophysical and physiological studies support the notion of neural circuits tuned to different interval durations (Nagarajan et al., 1998; Meegan et al., 2000; Bartolo and Merchant, 2009; Merchant et al., 2013; Bartolo et al., 2014). It is also known that timing different types of movement, biological vs. non-biological for example, is performed by different brain structures that can be selectively manipulated (Avanzino et al., 2015), and this is also consistent with the idea that there is no central general-purpose chronometer.

Our data shows that Weber fraction decreases exponentially as a function of elapsed time and that this is due to the $\sigma_{indep}^{2}$ term, that is, to the presence of a basal variability (y-intercept on the Std vs. *Go-time* graphs, Figure 4). As can be observed in the graphs, the effect of this basal variability reduces at longer elapsed times. However, a recent study in which Grondin and colleagues asked subjects to count at different speeds showed that Weber fraction increased in proportion to the interval length used to subdivide a large total time and that this effect persisted for elapsed times of up to 24 s (Grondin et al., 2015). Their results also showed that mean produced time was always shorter than real elapsed time. Contrary to Grondin's findings, our data predicts that no differences should be observed for long and short subdividing intervals when total elapsed times are larger than ~3 s and that errors should not be all negative but instead should be positive for long subdividing intervals and negative for short intervals. We suggest that these differences could be explained by differences in the experimental design. We used interleaved trials in which total elapsed time (*Go-time*) and interval length were pseudo-randomly selected while Grondin and colleagues used a blocked design in which subjects performed the trials of different subdividing intervals in separate sessions. We believe this is an important difference because it has been demonstrated that subjects adjust their internal chronometer according to the distribution of timing intervals they must estimate (Jazayeri and Shadlen, 2010). As was the case in Buonomano's task, subjects in Grondin's experiments had to count up to a predetermined total number of intervals, prompting them to measure total elapsed time.

It is well known that subdividing a long interval into smaller ones decreases the total variance of the estimated elapsed time. Although our task was not designed to explore this phenomenon our results show that subdividing total elapsed time into 0.5 s intervals reduces the timing variability as compared to subdividing with 1 s intervals. This can be observed in Figure 4C by comparing the variability of the red and blue lines. We note however, that the beneficial effect of subdividing elapsed time into 0.5 s intervals is limited to total elapsed time of 3–4 s.

It is known that macaque monkeys do not easily entrain to temporal rhythms and that training them in rhythmic tapping tasks might take up to a year (Zarco et al., 2009; Merchant and Honing, 2014; Patel and Iversen, 2014). We speculate that the spatial component of our visuospatial task was an important sensory element that helped the monkeys better perceive and maintain rhythms of different paces. There is evidence that macaques rely more on visual than on auditory cues to control their timing behavior (Zarco et al., 2009; Merchant and Honing, 2014). Nevertheless, the timing behavior of monkeys followed the same pattern of temporal variability and constant errors than humans in a synchronization-continuation tapping task (Zarco et al., 2009).

A possible alternative explanation is that monkeys did not engage the visuo-spatial rhythm but relied instead only on an association between elapsed time and target position. However, we consider this possibility unlikely. The association between *Go-times* and target position was not fixed. In the *2-choice* task, for example, the stimulus randomly initiates on the left or the right. In the *6-choice* task the stimulus randomly initiates in any of the 6 positions and can rotate either clock wise or counterclockwise. This variation in initial conditions (remember that interval length and *Go-times* are also selected pseudo-randomly) makes it highly unlikely that subjects were mapping a given *Go-time* with a fixed target position. We would like to mention that, although we only report the behavior in the 2-choice task, monkeys were initially trained in a version of the 6-choice task in which the interval length was chosen from a continuous distribution (300–1200 ms, uniform distribution). During this phase of training, the number of *presentation* intervals was also variable (1–4, uniform distribution). Thus, the stimulus position at any given elapsed time was dependent on (1) the position of the first *presentation* interval, (2) the direction of stimulus rotation, (3) the number of *presentation* intervals, (4) the interval length (chosen randomly from a continuous distribution), and finally (5) the *Go-time* itself. This variation in initial conditions makes it practically impossible for the monkeys to learn all possible *Go-time* and stimulus position combinations and instead encourages them to use the rhythmic motion of the stimulus to predict its future position once it is no longer visible (Coull and Nobre, 2008; Coull, 2009).

The neuronal mechanisms underlying our perception of time and our ability to predict periodic sensory events are not yet completely understood (Roux et al., 2003; Ivry and Spencer, 2004; Eagleman et al., 2005; Coslett et al., 2009; Coull et al., 2011; Wittmann, 2013). It is only recently that the physiological correlates of timing have begun to be systematically investigated in primates (Ghose and Maunsell, 2002; Leon and Shadlen, 2003; Janssen and Shadlen, 2005; Genovesio et al., 2006; Fiorillo et al., 2008; Lebedev et al., 2008; Mita et al., 2009; Machens et al., 2010). It is known that neuronal correlates of timing can be found in parietal, motor, and pre-motor cortices of the primate cerebral cortex (Roux et al., 2003; Merchant et al., 2011; Jazayeri and Shadlen, 2015). These studies revealed distinct groups of neurons whose activity dynamics correlate either with elapsed time from the last motor or sensory event, or with the time remaining to the next motor command.

It is our goal to contribute to the understanding of the neural mechanisms of time estimation and time reproduction. We developed the visuospatial timing task in non-human primates to use it as an experimental model for studying the neuronal correlates of timing. This rhythm task is an ideal experimental setting because it lacks any movement during the *continuation* phase and it will let us study the neuronal correlates of timing without interference by movement or sensory-related activity.

## Author contributions

Vd, OG, and HM conception and design of research; Vd and OG performed experiments; Vd, OG and JC analyzed data; Vd, OG, HM, and JC interpreted results of experiments; Vd prepared figures; Vd, HM, OG, and JC edited and revised manuscript; Vd drafted manuscript.

### Conflict of interest statement

The authors declare that the research was conducted in the absence of any commercial or financial relationships that could be construed as a potential conflict of interest. The reviewer AP and handling Editor declared their shared affiliation, and the handling Editor states that the process nevertheless met the standards of a fair and objective review.
